# A call for public archives for biological image data

**DOI:** 10.1038/s41592-018-0195-8

**Published:** 2018-10-30

**Authors:** Jan Ellenberg, Jason R. Swedlow, Mary Barlow, Charles E. Cook, Ugis Sarkans, Ardan Patwardhan, Alvis Brazma, Ewan Birney

**Affiliations:** 1 European Molecular Biology Laboratory, Heidelberg, Germany; 2 Division of Computational Biology, School of Life Sciences, University of Dundee, Dundee, UK; 3 European Molecular Biology Laboratory, European Bioinformatics Institute (EMBL-EBI), Wellcome Genome Campus, Cambridge, UK

**Keywords:** Imaging, Data publication and archiving

## Abstract

Public data archives are the backbone of modern biological research. Biomolecular archives are well established, but bioimaging resources lag behind them. The technology required for imaging archives is now available, thus enabling the creation of the first public bioimage datasets. We present the rationale for the construction of bioimage archives and their associated databases to underpin the next revolution in bioinformatics discovery.

## Main

Since the mid-1970s it has been possible to analyze the molecular composition of living organisms, from the individual units (nucleotides, amino acids, and metabolites) to the macromolecules they are part of (DNA, RNA, and proteins), as well as the interactions among these components^1–3^. The cost of such measurements has decreased remarkably over time, and technological development has widened their scope from investigations of gene and transcript sequences (genomics/transcriptomics) to proteins (proteomics) and metabolic products (metabolomics). However, life cannot be understood simply from measurements of the presence of biomolecules; scientists also need to know when and where these biomolecules are present and how they interact inside cells and organisms. This requires mapping of their spatiotemporal distribution, structural changes, and interactions in biological systems.

Observation and measurement of the ‘when’ and ‘where’ in living organisms predates chemical measurements by more than three centuries: such observation began with light microscopy^4^ and was augmented centuries later with diverse ‘new’ technologies such as electron microscopy, X-ray imaging, electron beam scattering, and magnetic resonance imaging. These direct physical measurements range from the atomic scale to the whole-organism scale and have a striking dual use: they provide quantitative measurements of molecular structure, composition, and dynamics across many spatial and temporal scales and, in parallel, powerful visual representations of biological structures and processes for the scientific community and the wider public. With the huge expansion of imaging at all levels made possible by revolutionary technologies including cryo- and volume-electron microscopy, super-resolution light microscopy, and light-sheet microscopy, the opportunities for research and biomedical insights have never been greater. Delivering on this potential requires open sharing of image data to encourage both reuse and extraction of knowledge.

Despite the long history of biological imaging, the tools and resources for collecting, managing, and sharing image data are immature compared with those available for sequence and 3D structure data. In the following sections we outline the current barriers to progress, the technological developments that could provide solutions, and how those infrastructure solutions can meet the outlined need.

Most important, “imaging” is not a single technology but an umbrella term for diverse technologies that create spatiotemporal maps of biological systems at different scales and resolutions (Table 1). This is further complicated by the large size and diversity of image datasets and the concomitant diversity of the associated computational analysis tools. Achieving the level of integration for imaging data that is routine in biomolecular data will require coordination of data resources and community approaches that harmonizes measurements across spatiotemporal scales, imaging modalities, and data formats. In specific areas, the biological imaging community has tackled a number of these challenges, as exemplified by coordinated data-format reading schemes^5^ and individual examples of harmonized measurements across scales; however, there is far more potential that could be achieved through data accessibility, reuse, and integration.

**Table 1:** Biological scales of imaging

Scale (unit)	Imaging technology	Use
Molecular (angstrom)	Single-particle cryo-electron microscopy (EM) and electron tomography averaging	Structural analysis, molecular function
Molecular machines (nanometer)	Cryo-EM, super-resolution light microscopy (SRM)	Biochemistry, molecular mechanisms
Cells (micrometer)	Transmission EM, volume EM, light microscopy (wide-field, confocal, SRM), electron tomography, 3D scanning EM, soft X-ray tomography	Cellular morphology, activity within cells, mechanism
Tissues (millimeter)	Volume EM, scanning EM, light microscopy (multiphoton, light sheet, OPT, etc.), X-rays (micro-CT), fluorescence imaging, mass spectrometry imaging	Protein localization, tissue morphology and anatomy, interactions between cells
Organism/organ (centimeter)	Photography, X-rays, magnetic resonance imaging, optical tomography technologies, computerized tomography, luminescence imaging	Mechanistic understanding of development and disease

Imaging is used to understand a range of phenomena at different size and time scales. In general, image capture at different scales uses different technologies and records different types of metadata.

Recent breakthroughs in imaging technology and in computer science now make it both feasible and of the utmost importance to address this challenge. First, the resolution revolutions in light and electron microscopy (recognized with the Nobel Prize in chemistry in 2014^6^ and 2017^7^) now routinely allow molecular identification and position and structure determination in imaging data from biological systems, rapidly bringing the where and when of molecular structure and function within reach. New advances in automation and throughput allow this to be done in a systematic manner. We expect that, for example, super-resolution imaging will be applied across biological and biomedical imaging; examples in multidimensional imaging^8^ and high-content screening^9^ have recently been reported. It is very likely that imaging data volume and complexity will continue to grow. Second, the emergence of powerful computer-based approaches for image interpretation, quantification, and modeling has vastly improved the ability to process large image datasets. These approaches are underpinned by the rapid evolution of algorithms, data storage, and cloud computing technologies. Wider adoption of such technology has also led to substantial decreases in cost, thus making the establishment of integrated public bioimage data resources feasible and highly valuable. We believe that the establishment of a biological image archive, with coordinated data resources brokering image data deposition and further analysis, will provide community momentum and a strong incentive to harmonize both measurements and analysis approaches.

## Data archives and added-value databases

There are two distinct types of biological databases. Data archives are long-lasting data stores with the dual goals of (1) faithfully representing and efficiently storing experimental data and supporting metadata, thus preserving the scientific record, and (2) making these data easily searchable by and accessible to the scientific community. An archive serves as an authoritative public resource for data, but it does not aim to synthesize datasets or make value judgments beyond assuring adherence to standards and quality. Archives make it possible to connect different datasets on the basis of common standardized elements, such as genes, molecules, and publications. A typical example of a biological data archive is the European Nucleotide Archive^10^, which stores nucleotide sequences. Data archives often have a single global scope, even if they are provided by a distributed organization worldwide.

The second type, referred to as added-value databases, are synthetic: they enrich and combine different datasets through well-designed analysis, expert curation, and, where possible, meta-analysis. They typically provide integrated information and biological knowledge for a community of users. They may also include advanced functionalities such as question-oriented searches and queries, cross-comparison of datasets, and advanced mining and visualization. Examples of added-value image databases exist already, such as the Image Data Resource^11^ (IDR; https://idr.openmicroscopy.org/), which integrates cell and tissue imaging studies on the basis of genetic or drug perturbations and phenotype, and PhenoImageShare^12^, which uses defined ontologies to integrate image datasets on the basis of phenotype.

We propose the creation of a central ‘BioImage Archive’ that would provide the foundation for existing and new future added-value databases. Data pipelines from this archive would feed databases, and at the same time maximize the possibilities for knowledge extraction and new discoveries by allowing rapid data aggregation and cross-discipline comparisons. Given the currently available and rapidly growing image datasets in the Electron Microscopy Data Bank (EMDB)^13^, Electron Microscopy Public Image Archive (EMPIAR)^14^ (Box 1), and IDR resources and beyond (Table 2), we see this archive as a response to an urgent community need, as well as an essential foundational layer for the accelerated development of new added-value resources.

**Table 2:** Examples of potential high-value datasets

Data type	Utility and impact	Types of users/applications	Examples of public resources
Correlative light and electron microscopy	Link functional information across spatial and temporal scales	Structural biologists and modelers: structural models that span spatial and temporal scales	EMPIAR^14^
Cell and tissue atlases	Construction, composition, and orientation of biological systems in normal and pathological states	Educational resources; reference for construction of tissues and/or organisms	Allen Brain Atlas (https://www.brain-map.org), Allen Cell Explorer (https://www.allencell.org/), Human Protein Atlas (https://www.proteinatlas.org), Human Protein Cell Atlas (https://www.proteinatlas.org), Mitotic Cell Atlas (https://omictools.com/mitotic-cell-atlas-tool), model-organism gene expression atlases^19^
Benchmark datasets	Standardized test datasets for the development of new algorithms	Algorithm developers, testing systems	EMDataBank^13^, BBBC (https://data.broadinstitute.org/bbbc), IDR^11^, CELL Image Library (http://www.cellimagelibrary.org)
Systematic phenotyping	Comprehensive studies of cell structure, systems, and response	Queries for genes or inhibitor effects	MitoCheck (http://www.mitocheck.org), SSBD (http://ssbd.qbic.riken.jp), IMPC (https://www.mousephenotype.org), PhenoImageShare^12^

This table is exemplary and is not a comprehensive survey of all imaging datasets.

A clear separation between data archives and added-value databases is important for ensuring the smooth flow of data from the experimentalists generating the data to the public archives. If journals make it a requirement that image data supporting a publication must be submitted to the BioImage Archive, the data-submission process must be straightforward and rapid, and should produce a unique identifier for citation of the data. Added-value databases, in contrast, may want to carefully curate the submitted data, and often require additional quality control, updates to ontologies, or reprocessing of the data. The separation of the archival layer from the added-value layer makes both functions possible, efficient, and effective.

**Fig. 1:**
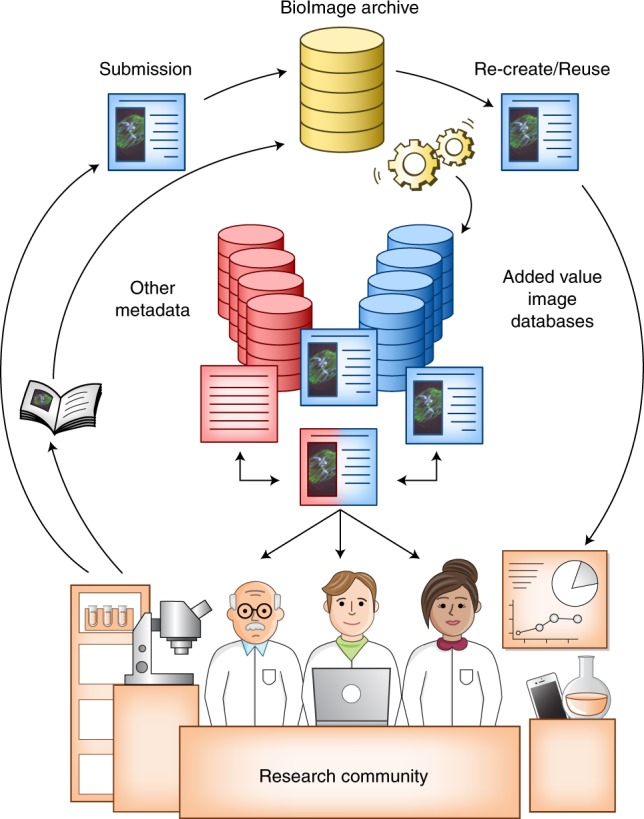
The bioimaging-data archiving ecosystem. The flow of data between the different parts of the community is shown. The BioImaging Archive serves as the central bioimage data repository for the scientific community, while added-value databases consume reference datasets and enable reuse and integration. Credit: Marina Corral Spence/Springer Nature.

Thus, a functional biological imaging data storage and coordinated data resource would include both archival and added-value aspects (Fig. 1). The archival repository of images would preferably be supported by an international collaborative effort with common standards in the same way that DNA archives are part of the International Nucleotide Sequence Database Collaboration consortium (http://insdc.org)^10^. Added-value databases, developed around the archive, would focus on particular biological areas where greater understanding can be obtained through systematic integration of images, just as the Ensembl database and genome browser^15^ add value to DNA data, and the Expression Atlas^16^ integrates archived data to elucidate gene expression in a biology-centric way.

## The rationale for a BioImage Archive

The BioImage Archive should store and make available imaging datasets from the molecular to the organism scale (Table 1). Archived datasets should be directly related to the results and figures included in a publication. Scaling of such an archive to the large number of publications that contain bioimage data is made possible by new, efficient, object-based storage systems. A key aspect for the sustainability of large-scale data archiving has been the deployment of technologies to ensure that the growth in the volume of data year to year matches the decrease in disk storage costs; for DNA sequences, this has required the development of data-specific compression. Similar methods of highly effective lossless or near-lossless compression and big-data handling are currently being developed for digital images^17,18^.

To accelerate the development of the BioImage Archive, datasets that are likely to be reused and of high value to the community should be rapidly identified. So-called reference images, a concept that was introduced in a white paper published jointly by Euro-BioImaging (https://www.eurobioimaging.eu), the European infrastructure for imaging technologies and ELIXIR (https://www.elixir-europe.org), and the European infrastructure that coordinates life science data resources (https://www.eurobioimaging.eu/content-news/euro-bioimaging-elixir-image-data-strategy), are data that have value beyond a single experiment or project because they also can serve as a resource for the larger community. They must be interpretable by researchers outside the laboratory that generated them and also of general interest for many biologists. Examples of such images are electron microscopy maps of protein structure, systematic characterizations of proteins in a common cell type, mapping of spatiotemporal expression patterns or phenotypes, and organ or developmental atlases. Such reference images not only will be useful to the research community, but also would serve to demonstrate the value of bioimage data archiving. At the beginning, defining the principles for the identification of reference-image datasets will require a scientific review process. Over time, however, image datasets would be expected to attain a ‘highly used’ label based on their actual reuse, similar to citation scores of scientific publications.

The scope of the archive would not be tied to any particular imaging technology; rather, it would be defined by the life sciences community on the basis of the likelihood of data reuse. This is an established guiding concept in biomolecular data archiving; for instance, the Gene Expression Omnibus and the Functional Genomics Data Archive ArrayExpress^19,20^ accept data from different experimental modalities and generated by different technologies. The acceptance is based on the data’s relevance to functional genomics, which in practice is defined by the goals of the project that generated the data or by the journal where the research paper is published.

We list below four key synergistic functions that the integrated ecosystem of a bioimage data archive and associated added-value databases should fulfill:

1. Promote open data and reproducibility of research, following the FAIR principles^21^, allowing authors to provide a full audit trail of their original image data and analysis methods, and allowing other interested scientists to explore alternative analyses of the same raw data. Making data, materials, and methods used in scientific research available to other researchers, with no restrictions on their use other than a request for a citation, is a long-standing tradition in the life sciences, as well as an essential bedrock principle of scientific progress and rigor^22,23^.
2. Provide reference data for the research community. These data, provided with rules of community use and analysis methods, can prevent the redundant production of replica datasets and serve as the basis for added-value resources (e.g., atlases). They improve the efficiency of routine experiments and over time will constitute a comprehensive scientific reference-image resource for cells, tissues, organs, and organisms.
3. Allow new scientific discoveries to be made with existing data, in particular via novel or integrated analysis of datasets that were originally generated for a different purpose or as a resource. The feasibility and value of such reanalysis of combined imaging datasets has already been demonstrated^24^. Integrative structure determination is another example of such a synergy^25^.
4. Accelerate the development of image-analysis methods using a broad range of benchmark datasets, thus encouraging validation and robustness of these computational methods and enabling well-structured image-analysis challenges^24,26,27^.

A similarly strategic approach to data archiving has been critical to the success of genomics—indeed, it has amplified the value of DNA sequencing data enormously, as archives allow the comparison of new sequences to all previously archived sequences. Although we recognize that biological image data are more complex, context dependent, and multidimensional, such dependencies are not entirely new for molecular archives. Gene-expression data, for example, are also context dependent, multidimensional, and potentially affected by experimental conditions. Three-dimensional macromolecular structures and electron microscopy data have been archived for 45 and 15 years, respectively, and this has proven to be tremendously valuable^28–30^. A central BioImage Archive will prove just as powerful.

## Paying for a BioImage Archive

Economic analysis of EMBL-EBI’s biomolecular data resources^31^ shows that research efficiency is increased many-fold by the availability of reference data (function 2)—enough to more than compensate for the storage and running of these resource—and that only open data enable assessment of reproducibility (function 1). An integrated archive will provide a long-term home for image data that has high utility and reduces the need for every institution with substantial image-data-generation capabilities to invest in long-term archiving efforts. For consortia and projects planning to systematically generate large amounts of imaging data, the archive would become a trusted partner in presenting cost-effective and sustainable solutions for image deposition and storage to their funders. As with biomolecular data resources, the maximum utility is realized when all scientists (whether in academia or industry) can freely deposit to the archive and freely access and reuse the data. This will be more and more the standard case, as many research funders now include mandatory data-management plans in their grants, for which the archive would again be the trusted partner.

We have considered the concern that a BioImage Archive would be flooded by enthusiastic depositors offloading their storage costs to the central resource without scientific benefit for anyone beyond the submitting group. Our experience of running DNA archives is that the requirement that datasets be associated with a publication and the inclusion of key quality control steps and management prevent ‘spurious’ use of the archive. Importantly, the BioImage Archive would not replace the need for initial local storage for capture, quality control, and primary image analysis in scientific projects; it would, however, provide long-term archiving after publication. As noted above, we propose to start filling the archive with reference-image datasets, but it is quite likely that the definition and scope of reference images will evolve as the archive grows and its associated databases develop protocols and tools for identifying and integrating data from the archive. An evolution of data-submission standards has occurred with other archives, and discussions with the imaging community at an international workshop we convened (details are included in the Acknowledgements) suggest a similar dynamic in the bioimaging community.

## Easy submission of image data will drive the value of the archive

One of the keys to successful sharing is the ease of submitting data to the archives. Submission criteria must balance the collection of standardized data and metadata necessary for dataset interpretation against the risk of overburdening submitters with complex technical requirements. Moreover, it is not always obvious what metadata are minimally required, particularly when dealing with rapidly evolving technologies like imaging. The solution to this problem is for relevant scientific communities to rapidly agree on the initial minimum requirements and update them as technologies change and science advances, for example, as in the case of the Genomic Standards Consortium (http://gensc.org), which works to develop community-driven ‘minimum information’ standards for descriptions of genomes, metagenomes, and marker genes. However, with the rapid advances in imaging technology, the underlying data structures must also evolve with changing requirements, and the submission tools will have to coevolve to keep up with those changes.

Additionally, journals and funding agencies should exert their influence to encourage data submission. The BioImage Archive should work closely with journals and preprint servers to encourage image-data deposition at the time of submission or acceptance for publication. The image-data sharing infrastructure must demonstrate a positive return on investment to both funders and data submitters. The involvement of funders will be crucial to ensure that large systematic data-generation projects commit to depositing their image data and appropriately budget for these costs in projects.

The ultimate driver for data submission will be scientific impact: sharing and reuse of data maximize the impact of research, and can increase the volume of citations by one or two orders of magnitude^32^. The BioImage Archive will ensure the preservation of the scientific record while building up a critical mass of reusable data. Associated added-value databases, as noted above, will build on the archive to provide in-depth curation, annotation, standardization, reanalysis, and integration of independent datasets.

## Human image archiving

There are many potential synergies between the use of imaging in clinical research and practice and that in basic biological research. Both domains require robust, usable tools for data processing and analysis, and there are many opportunities for reuse of methods, algorithms, and software. Because of the extensive use of imaging in clinical research and practice, there is also an increasing need to link imaging used for human phenotyping with genotyping efforts, such as in the UK BioBank^33^. Although there are analogies to human genome data sharing between researchers (for example, as enabled by the European Genome-phenome Archive (https://www.ebi.ac.uk/ega)), data sharing in the clinical community has different challenges and a different culture. Additionally, practical factors such as appropriate consent, ethics approval, and data privacy must be addressed before broader sharing protocols can be set up. Our recommendation is to use the BioImage Archive to drive concrete, example-driven discussions and recommendations around these issues. We aim to (a) continue bringing the atomic, cellular, tissue, and model organism imaging communities together with the clinical community; (b) continue a broad discussion on the merits and requirements of comprehensive data sharing among clinical researchers, with a particular focus on factors of known complexity, such as consent and privacy; and (c) where possible, incorporate suitably anonymized and consented clinical image datasets into the BioImage Archive to help establish the correct submission requirements and overall value of these data. These submissions may come directly from study authors or eventually from applications such as i2b2 that have been adapted to submit data to the BioImage Archive’s submission portal^34^.

## Building a bioimage database system and its user communities

We envisage an integrated BioImage Archive with user-friendly submission systems that support deposition using community-developed data standards, interconnected with a growing and diverse set of added-value databases that together evolve into a comprehensive bioimage database system. We have already made progress toward this end with the establishment of the BioStudies archive and IDR. BioStudies enables authors to package all data supporting a publication, either by providing links to data in specialized databases such as the Protein Data Bank (PDB) or by storing the actual data when a dedicated resource does not exist^35^. It has a flexible structure that supports metadata capture from rapidly evolving technologies, including imaging. While the standards for bioimaging data submission and a dedicated archive are being developed by the relevant community, BioStudies fulfills the role of a temporary bioimage data archive; its ‘working blueprint’ approach will help developers design functionality for imaging data submissions and access, determine practical and sufficient metadata requirements, and build a scalable technical infrastructure capable of dealing with large data volumes. We are currently working to connect BioStudies and the IDR so that reference-image data in BioStudies can be easily imported into IDR for inclusion in the added-value resource. At the time of writing, before the establishment of the BioImage Archive, these two resources held nearly 5,000 bioimage datasets and 2.9 million individual images. These developments are an exemplar for the coordination needed to deliver the overall vision: develop submission protocols, establish review processes for identifying reference datasets, assemble imaging datasets, and build the publication systems that will be the foundation for the mature bioimage database system.

The mature BioImage Archive, like those already established for genomics, transcriptomics, and proteomics, will need to be a large international effort, leveraging contributions, resources, and technology from the global scientific community. In Europe, the Euro-BioImaging consortium provides a framework for imaging-research infrastructure. During Euro-BioImaging’s planning, representatives from almost all EU member states participated in user surveys, technological evaluations, and proof-of-concept tests of a transnational bioimaging infrastructure. This activity produced plans for image-data archiving and community-accessible tools for image analysis and processing. Euro-BioImaging has recently submitted its application to become a European Research Infrastructure Consortium to obtain independent international status and implement the plans developed in the preparatory phase.

Euro-BioImaging, the IDR, and the strategic collaboration with ELIXIR are examples of how national and transnational efforts can collaborate to deliver the components of the bioimage data ecosystem we have outlined here. Past experience with resources such as the PDB demonstrates that transnational initiatives that include scientists, funders, and institutions are best placed to build and sustain community data resources. Indeed, given that the proposed resources will fuel life science research globally and not just in Europe, a worldwide community might ultimately contribute to their long-term operation. Connecting European efforts with international partners is a major objective of the Global BioImaging Project (http://www.eurobioimaging.eu/global-bioimaging). Euro-BioImaging, Global BioImaging, and other national and transnational imaging networks can serve as powerful advocates for the adoption and use of the bioimage data ecosystem by scientists, institutions, journals, and funders. This is already beginning: as of this writing, Springer Nature is recommending that its authors use IDR for their imaging data.

## Conclusions

Three parallel developments have together created both the need for an open access BioImage Archive and the opportunity to establish it: (1) the development of new imaging technologies that generate large, high-quality image datasets ranging from molecular and structural information to organismal descriptions; (2) faster computers and new computational methods that allow faster, better analysis of these images; and (3) the decreasing costs of data storage and cloud computing technologies. The possibilities for deeper biological understanding opened up by new imaging technology make the development of the BioImaging Archive and the corresponding coordinated data resources critical for new opportunities in research, methods development, and training. To deliver on the promise of imaging technology, it is essential that scientists openly share biological image data.

We envision a BioImage Archive that will store reference images that are freely available for reuse. These are very likely to include any image that has been formally published, as well as other, curated image datasets. The archive will, in turn, support a host of added-value image-data resources that will enhance the scientific value of the archival images through curation, integrative analysis, and the development of new analytical methods.

The practical experience of the latest generation of bioimaging data resources shows that data volumes can be technically managed at scale and, importantly, that datasets generated by the latest imaging technologies can be made FAIR. Examples of reuse of bioimaging data suggest that the long-term value of these datasets, just like that of public biomolecular data, will grow and evolve. The construction of public bioimage data resources is just beginning, and we need to continue to expand engagement across bioimaging communities, in particular in clinical imaging, as well as with scientific journals and funders. Nevertheless, it is already clear that routine bioimage archiving will deliver strong scientific and economic returns, as shown by the reuse made possible by the EMPIAR and IDR resources. The potential resulting research discoveries and clinical applications make the integration of the proposed BioImage Archive and associated added-value databases an imperative for the biological, computational, and, in the future, clinical research communities.

Box 1 Archives for the EM revolution One prominent public imaging resource is in the area of structural biology. Increased detector sensitivity and new methods in electron microscopy have created a new approach to atomic-scale measurement of biological molecules: single-particle cryo-electron microscopy. This method bridges the gap between the atomic resolution of crystallography and that of diffraction-limited light microscopy to provide visualization of large protein complexes at near-atomic resolution.

The structural biology community has enthusiastically embraced these technologies, implementing resources for cryo-electron microscopy imaging (EMDB (http://emdb-empiar.org/), established at EMBL-EBI in 2002, and EMPIAR (http://empiar.org/), established at EMBL-EBI in 2014) that robustly demonstrate the value of image resources^13,14^. EMDB now has more than 6,000 entries, with one-third of these released in the past two years, and this growth is mirrored by a rapid rise in the number of unique hosts accessing the service, which grew 20-fold to almost 100,000 between 2009 and 2017.

This rapid growth is unlikely to slow. In the UK alone, the number of high-end microscopes will increase from 7 in 2016 to nearly 20 in 2018. Worldwide, 50 Titan Krios microscopes were installed between 2008 and 2015, around 100 were installed in 2016–2017, and even more will come online in 2018 (S. Welsh, personal communication).
